# Supplementary material for: An Innovative Arteriovenous (AV) Loop Breast Cancer Model Tailored for Cancer Research
Source: Bioengineering (Basel). 2022 Jun 27;9(7):280. doi: 10.3390/bioengineering9070280 (PMC9311974; doi:10.3390/bioengineering9070280)
Supplement: Supplementary file 1 [file bioengineering-09-00280-s001.zip › bioengineering-1742407-supplementary.pdf]

bioengineering

Supplementary Materials

An Innovative Arteriovenous (AV) Loop Breast Cancer Model Tailored for Cancer Research

Ran An [1,2], Pamela L. Strissel [3], Majida Al-Abboodi [1,4], Jan W. Robering [1,5], Reakasame Supachai [6], Markus Eckstein [7], Ajay Peddi [1,8], Theresa Hauck [1], Tobias Bäuerle [9], Aldo R. Boccaccini [6], Almoatazbellah Youssef [10,11], Jiaming Sun [2], Reiner Strick [3], Raymund E. Horch [1], Anja M. Boos [1,5] and Annika Kengelbach-Weigand [1,*]

1 Department of Plastic and Hand Surgery, Laboratory for Tissue Engineering and Regenerative Medicine, University Hospital Erlangen, 91054 Erlangen, Germany; an_ran2018@163.com (R.A.); majida.al-abboodi@fau.de (M.A.-A.); jrobering@ukaachen.de (J.W.R.); ajay.pharma20@gmail.com (A.P.); theresa.hauck@uk-erlangen.de (T.H.); raymund.horch@uk-erlangen.de (R.E.H.); aboos@ukaachen.de (A.M.B.)
2 Department of Plastic Surgery, Union Hospital, Tongji Medical College, Huazhong University of Science and Technology, Wuhan 430022, China; an_ran2018@163.com; sunjm1592@sina.com
3 Department of Obstetrics and Gynecology, University Hospital Erlangen, 91054 Erlangen, Germany; strisspa@outlook.com (P.L.S.); reiner.strick@uk-erlangen.de (R.S.)
4 Institute of Genetic Engineering and Biotechnology, University of Baghdad, 10081 Baghdad, Iraq
5 Department of Plastic- and Hand Surgery, University Hospital RWTH Aachen, 52074 Aachen, Germany
6 Institute of Biomaterials, Friedrich-Alexander University Erlangen-Nürnberg, 91056 Erlangen, Germany; supachai.reakasame@gmail.com (R.S.); aldo.boccaccini@ww.uni-erlangen.de (A.R.B.)
7 Institute of Pathology, University Hospital Erlangen, 91054 Erlangen, Germany; markus.eckstein@uk-erlangen.de
8 Institute of Clinical Radiology, University Hospital Münster, 48149 Münster, Germany
9 Preclinical Imaging Platform Erlangen (PIPE), Department of Radiology, University Hospital Erlangen, 91054 Erlangen, Germany; tobias.baeuerle@uk-erlangen.de
10 Department of Functional Materials in Medicine and Dentistry, University of Würzburg, 97080 Würzburg, Germany; moataz.youssef@fmz.uni-wuerzburg.de
11 Institute of Pathology, University of Würzburg, 97080 Würzburg, Germany
* Correspondence: annika.kengelbach-weigand@uk-erlangen.de

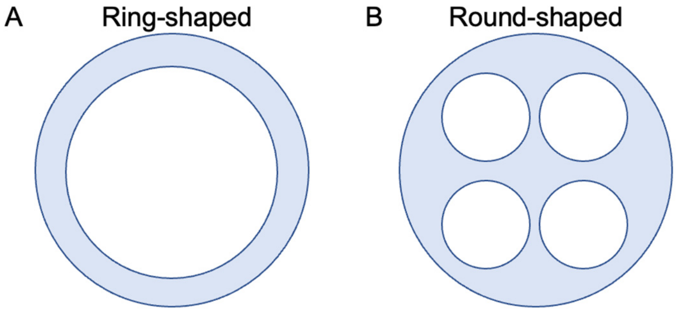

Figure S1. Schematic diagram of the ring- and round-shaped constructs.

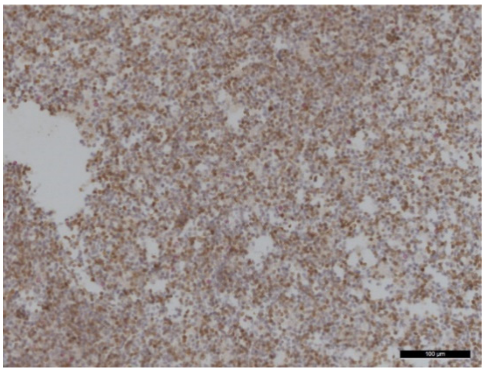

Figure S2. Representative image of CD3 immunohistochemical staining on rat blood cells. Scale bar 100 μm.

Table S1. Construct compositions and cell numbers for *in vivo* implantation.

	Construct composition	HTB-26 cell number
Control	alginate-fibrin capsules	none
Alginate-fibrin	alginate-fibrin capsules	5×10^6
PCL	6 x PCL scaffolds, alginate-fibrin	3×10^5 /scaffold 1 week before implantation
Fibrin	Fibrin	5×10^6
